# Exploring the role of ferroptosis-related genes as biomarkers in acute kidney injury

**DOI:** 10.1371/journal.pone.0307472

**Published:** 2024-07-23

**Authors:** Gang Luo, Yi Gao, Ziyu Zheng, Baobao Gao, Lini Wang, Xuemiao Tang, Chong Lei

**Affiliations:** 1 Department of Anesthesiology and Perioperative Medicine, Xijing Hospital, The Fourth Military Medical University, Shaanxi, China; 2 Shaanxi University of Chinese Medicine, Xianyang, Shaanxi, China; Jinan University, CHINA

## Abstract

**Introduction:**

Acute kidney injury (AKI) is a severe condition with high morbidity and mortality. Innovative biomarkers and treatments are essential for improving patient outcomes. This study aims to investigate the role of ferroptosis-related genes (FRGs) in AKI for identifying potential biomarkers and therapeutic targets.

**Methods:**

We analyzed mRNA expression profiles from the Gene Expression Omnibus (GEO: GSE139061) dataset, comparing 36 AKI samples with 9 normal samples. Differentially expressed genes (DEGs) were identified using the R software package limma. Functional enrichment analyses were conducted using Gene Ontology (GO) and Kyoto Encyclopedia of Genes and Genomes (KEGG) pathways. Key biomarkers were validated through area under the curve (AUC) values, and immune cell infiltration was analyzed using CIBERSORT.

**Results:**

We identified 78 differentially expressed FRGs, with 27 up-regulated and 51 down-regulated genes. Key signaling pathways included MAPK, ferroptosis, and p53. Five genes—*NR4A1*, *GLRX5*, *USP35*, *AEBP2*, and *MDM4*—were identified as potential biomarkers, each demonstrating AUC values greater than 0.800. Specifically, *MDM4* showed significant potential by promoting the phosphorylation of p53 at Ser46, enhancing mitochondrial apoptotic activity. Immune analysis revealed a significant elevation of M0 macrophages in AKI samples compared to normal samples (*P* < 0.01).

**Conclusion:**

Our findings highlight the critical role of ferroptosis-related genes in AKI, identifying *NR4A1*, *GLRX5*, *USP35*, *AEBP2*, and *MDM4* as key biomarkers with high diagnostic potential. These results provide novel insights into the molecular mechanisms of AKI.

## Introduction

Acute kidney injury (AKI) is a potentially life-threatening clinical syndrome associated with increased short-term morbidity and mortality [1]. Moreover, AKI elevates the risk of chronic kidney disease and end-stage renal disease [2]. Currently, standard diagnostic methods for AKI involve monitoring urinary output and serum creatinine concentration (sCr) [3]. However, sCr has limitations due to factors such as muscle mass, intravascular volume, assay interference, and drug interactions, leading to fluctuations in measured sCr levels and delayed AKI detection [4]. Thus, there is an urgent need for biomarkers that enable early detection of AKI.

Ferroptosis, a novel form of programmed cell death, is characterized by iron-dependent lipid peroxidation, mitochondrial atrophy, and increased mitochondrial membrane density. This process is regulated by specific genes and can be inhibited by iron-chelating agents and lipid peroxidation inhibitors [5–7]. Studies in animal models of folic acid-induced AKI have shown that Ferrostatin-1 (FER-1), a specific inhibitor of ferroptosis, can significantly improve renal function and reduce tissue damage [8–10]. This discovery raises a compelling question: Could targeting ferroptosis be a new therapeutic strategy for AKI? Additionally, Hepcidin, a key regulator of iron levels, has been shown to protect against AKI by reducing oxidative stress [11,12]. GPX4, the sole enzyme capable of reducing esterified oxidized fatty acids and cholesterol hydroperoxides, was studied by inducing GPX4-deficient mice. Within two weeks after GPX4 deletion, the mice experienced massive renal tubular cell death and acute renal failure [13,14].

Combining ferroptosis inhibition with iron regulation presents an innovative approach to mitigating AKI effects. Our study explores the expression of FRGs in AKI, aiming to identify biomarkers for early diagnosis and potential treatment targets. By understanding these molecular pathways, we hope to provide novel insights into AKI development and highlight new therapeutic possibilities. This research not only seeks to identify better diagnostic markers but also investigates promising treatment strategies, paving the way for advancements in AKI management.

## Materials and methods

### Microarray dataset collection and data processing

The microarray gene expression data used in this study were downloaded from the Gene Expression Omnibus (GEO) database. We obtained a comprehensive list of ferroptosis-related genes from FerrDb (http://www.zhounan.org/ferrdb/index.html). A total of 254 ferroptosis-related genes were collected and are provided in S1 Table.

### Differentially expressed gene analysis

The R software package limma was used to conduct differential analysis on 36 disease and 9 normal samples of GSE139061. The limma software package employs the classic Bayesian data analysis to screen DEGs. The significance criteria for DEGs were set at a *P* value of less than 0.05 and log Fold Change (logFC) greater than 1.5. The heatmap software package was used to draw the heatmap of DEGs and the R package clusterProfiler facilitated functional annotation.

### Identification of optimal diagnostic gene biomarkers for AKI

Our study used five machine learning algorithms, including Least Absolute Shrinkage and Selection Operator (LASSO), Support Vector Machine-Recursive Feature Elimination (SVM-RFE), Random Forest, Logistic Regression, and Decision Trees, to identify and validate the most effective diagnostic gene biomarkers for AKI. LASSO and SVM-RFE were selected as the most suitable methods due to their superior performance in distinguishing AKI samples from normal samples. The 8 disease-related features were selected using LASSO, which enhances model interpretability by focusing on significant predictors. The 9 disease-related features were identified using SVM-RFE, which improves model accuracy by iteratively refining the feature set. While there is some overlap between these two sets, each method also identified unique features, ensuring a comprehensive and robust selection of biomarkers for early detection and accurate diagnosis of AKI. This dual approach captures a balanced set of features, contributing to the reliability of our findings.

### Functional enrichment analysis

To perform functional enrichment analysis, we conducted Gene Ontology (GO) and Kyoto Encyclopedia of Genes and Genomes (KEGG) pathway enrichment analyses using the clusterProfiler package in R. GO analysis included three categories: Biological Process (BP), Cellular Component (CC), and Molecular Function (MF). KEGG pathway analysis provided insights into the involvement of differentially expressed genes in various biological pathways. Both analyses were performed using a hypergeometric test with a significance threshold set at adjusted *P* < 0.05. The results were visualized using ggplot2, depicting the enriched GO terms and KEGG pathways for the differentially expressed genes.

### Single gene Set Enrichment Analysis (GSEA)

The GSEA (V.4.1.0) package in R was utilized to investigate the related pathways of the seven marker genes. We calculated the correlation between these marker genes and all other genes in the GSE139061 dataset. Based on these correlations, all genes were sorted in descending order and considered as the gene set to be tested. Furthermore, we used the KEGG signaling pathway set as a predefined gene set because it provides a comprehensive and well-annotated resource for understanding the biological pathways involved in the gene set. This allows us to evaluate the enrichment of our identified genes within known biological pathways, facilitating the interpretation of their functional roles.

### Immune infiltration analysis

The CIBERSORT software was employed to predict the proportions of 22 distinct types of infiltrating immune cells in each tissue sample from the GSE139061 dataset. For each sample, the sum of the fractions corresponding to all evaluated immune cell types equaled one, ensuring a comprehensive assessment of immune cell proportions.

### Renal ischemia-reperfusion Model, RNA extraction, and qRT-PCR analysis

All experimental procedures were conducted following the "Guide for the Care and Use of Laboratory Animals" of the National Institutes of Health and ARRIVE guidelines. The ischemia/reperfusion-induced AKI mouse models were constructed according to previously published protocols [15]. 12-week-old male mice were divided into two groups: a sham-operated group and an ischemia-reperfusion injury (IRI) model group. Both groups acclimated for seven days under the same conditions. To induce AKI, we anesthetized the mice with 1.4% isoflurane delivered with 100% oxygen at a rate of 1.0 L/min and made a midline incision to expose the kidneys [16]. We then clamped the renal arteries and veins using vascular clamps, confirming successful clamping by observing the kidneys change from bright red to dark purple. After 60 minutes, we released the clamps, and the kidneys returned to their bright red color, indicating successful reperfusion. The sham-operated group underwent the same procedure without clamping [17]. For postoperative analgesia, we applied Eutectic Mixture of Local Anesthetics (EMLA) cream, which contains 2.5% lidocaine and 2.5% prilocaine [18]. Following surgery, the mice were placed on a heating pad for two hours and monitored until they fully awoke. They were then returned to their cages with free access to food and water.

Primer sequences for *NR4A1*, *GLRX5*, *USP35*, *AEBP2*, *MDM4*, and *GAPDH* gene were synthesized using the PrimerQuest (http://sg.idtdna.com/Primerquest) (Table 1). Total RNA was extracted using an RNA extraction kit (Hifair® Ⅲ 1st Strand cDNA Synthesis SuperMix for qPCR with gDNA digester), and reverse transcription was performed using the 11141es60 reverse transcription kit (Hieff® qPCR SYBR Green Master Mix with Low Rox Plus). The expression level of RNA, normalized to GAPDH, was calculated using the comparative Ct method (2-ΔΔCT).

**Table 1:** The primer sequences of NR4A1, GLRX5, USP35, AEBP2, MDM4 and GAPDH.

Gene name	Primer	Sequence(5’-3’)
MDM4	Forward primer	GAGCAGAAAGCTGAAACAGAAAG
	Reverse primer	GGCTCGTCTTCCCATGAATAA
AEBP2	Forward primer	TGAACAAGCGGAGGAAACTAA
	Reverse primer	GCAGATGGCTCGATGTCTTAT
USP35	Forward primer	GACCTCACAGAAGAGTGAACTG
	Reverse primer	GCATGTGTTGCCCAAGTTAAT
GLRX5	Forward primer	GGAGCTGAGGCAAGGTATTAAA
	Reverse primer	TGCATCTGCAGAAGGATGTC
NR4A1	Forward primer	TTATCCGAAAGTGGGCAGAAA
	Reverse primer	CACCGGGTTTAGATCGGTATG
GAPDH	Forward primer	AACAGCAACTCCCACTCTTC
	Reverse primer	CCTGTTGCTGTAGCCGTATT

### Ethical approval

The experimental protocols were approved by the Ethics Committee for Animal Experimentation at the Xijing Hospital, Fourth Military Medical University, Xi’an, China. All the experiments were conducted according to the Guidelines for Animal Experimentation of the Fourth Military Medical University.

Male C57BL/6 mice, aged 8–12 weeks, were provided by the Experimental Animal Center of the Fourth Military Medical University. All mice were housed under a 12-h light/dark cycle (lights on from 7:00 to 19:00), 23 ± 1°C temperature, 38–42% humidity, and free access to water and food.

### Statistical analysis

All statistical analyses were performed using R software (version 4.1.3), and graphs were visualized using the ’ggplot2’ package. Receiver operating characteristic (ROC) curve analysis and area under the curve (AUC) calculations assessed the diagnostic accuracy of gene expression levels for AKI. A significance level of *P* < 0.05 (two-sided) was considered statistically significant.

## Results

### Differential Expression Analysis (DEGs)

**Fig 1:**
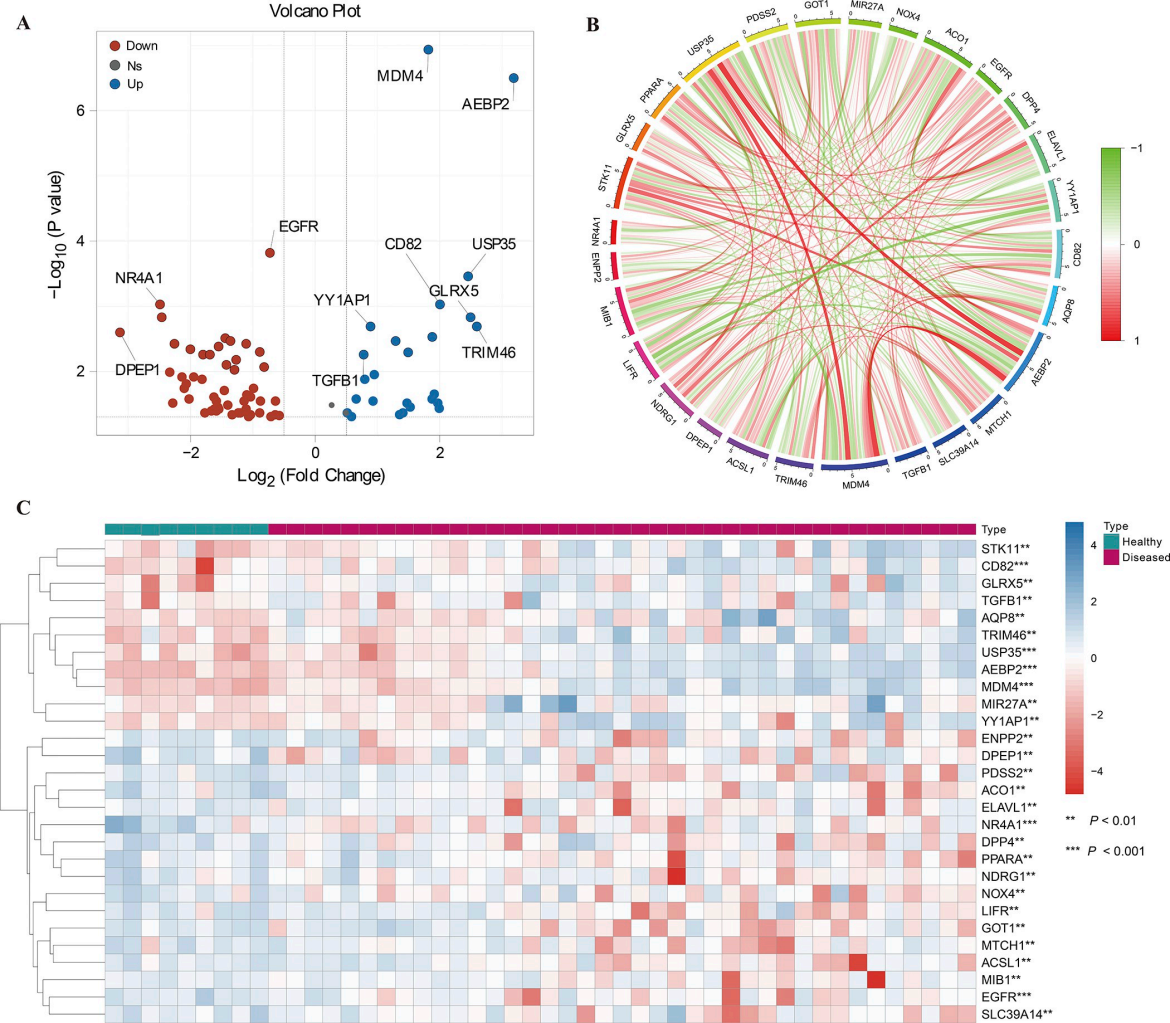
DE-FRGs expression levels in AKI. (A) Volcano map show expression patterns of DE-FRGs. (B) The correlation of DE-FRGs. (C) DE-FRGs expression profiles in AKI.

Seventy-eight of 237 FRGs were differentially expressed between AKI and normal samples, including 27 up-regulated and 51 down-regulated genes identified from the GSE139061 dataset (S2 Table). The Volcano plot displayed the expression pattern of 28 differentially expressed ferroptosis-related genes (DE-FRGs) (*P* < 0.001) among samples (Fig 1A). The correlation between these genes was presented in Fig 1B, showing USP35’s positive correlation with *MDM4* and *AEBP2*. *MDM4* had a positive correlation with *AQP8*, *STK11*, and *AEBP2*. The heatmap showed the expression profile of 28 DE-FRGs (*P* < 0.001) among samples. *STK11*, *CD82*, *GLRX5*, *TGFB1*, *AQP8*, *TRIM46*, *USP35*, *AEBP2*, *MDM4*, *MIR27A*, *and YY1AP1* were highly expressed in AKI samples (Fig 1C).

### Functional and pathway analyses of DE-FRGs

**Fig 2:**
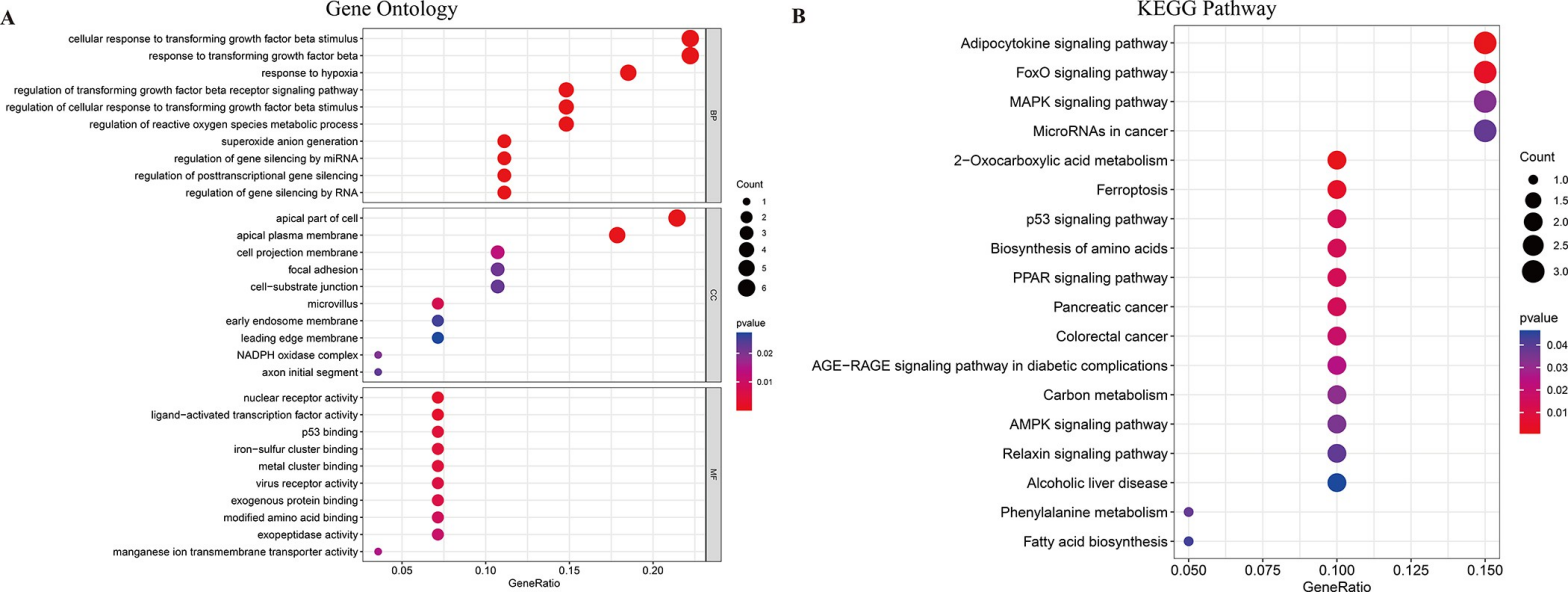
Functional analyses for the DE-FRGs. (A) GO enrichment analyses. (B) Pathway analyses.

To elucidate the biological roles of DE-FRGs in AKI, we performed GO enrichment and KEGG pathway analyses. GO enrichment analyses revealed significant associations of DE-FRGs with functions such as ’cellular response,’ ’apical part of cell,’ and ’regulation of reactive oxygen species metabolic process’ (Fig 2A). KEGG pathway analyses indicated that these genes are involved in critical signaling pathways, including Adipocytokine signaling, FoxO signaling, Ferroptosis, p53 signaling, Biosynthesis of amino acids, and MAPK signaling (Fig 2B). These findings suggest that DE-FRGs play crucial roles in the pathogenesis of AKI, particularly through mechanisms involving ferroptosis and cytokine regulation.

Further, single-gene GSEA-KEGG pathway analysis identified the top 10 pathways enriched for each marker gene (S1A–S1E Fig). These marker genes were significantly associated with biological processes such as ribosome function, autophagy, lysosome activity, cell cycle regulation, and immune response. The comprehensive analysis underscores the multifaceted roles of these genes in AKI, providing a deeper understanding of the molecular mechanisms underpinning the disease and highlighting potential targets for therapeutic intervention.

### Construction of the ferroptosis-related gene biomarkers in AKI

**Fig 3:**
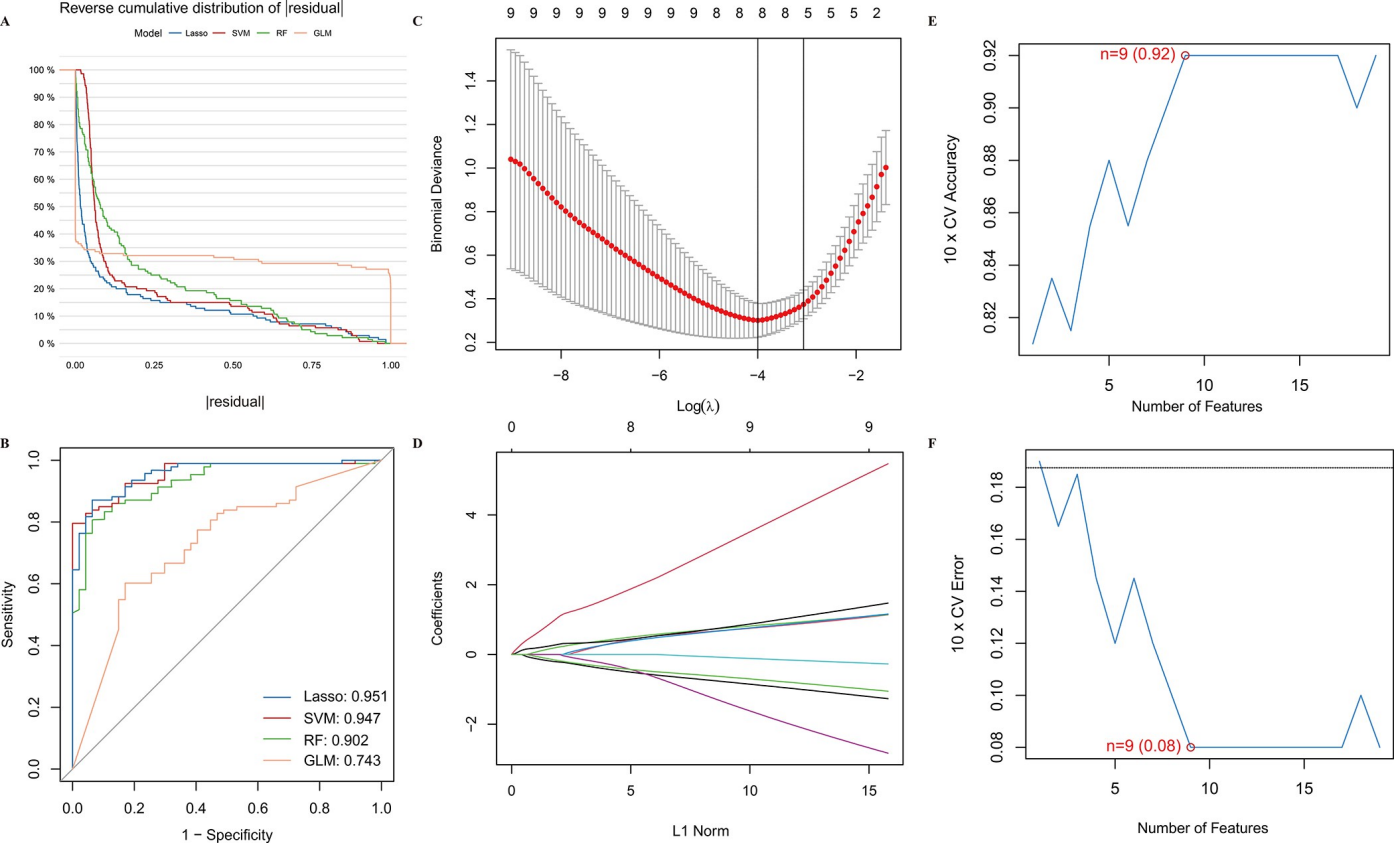
DE-FRGs were identified as diagnostic genes for AKI. (A, B) Model Selection. (C, D). Lasso algorithm to filter the DE-FRGs. (E, F). SVM-RFE algorithm to filter the DE-FRGs.

**Fig 4:**
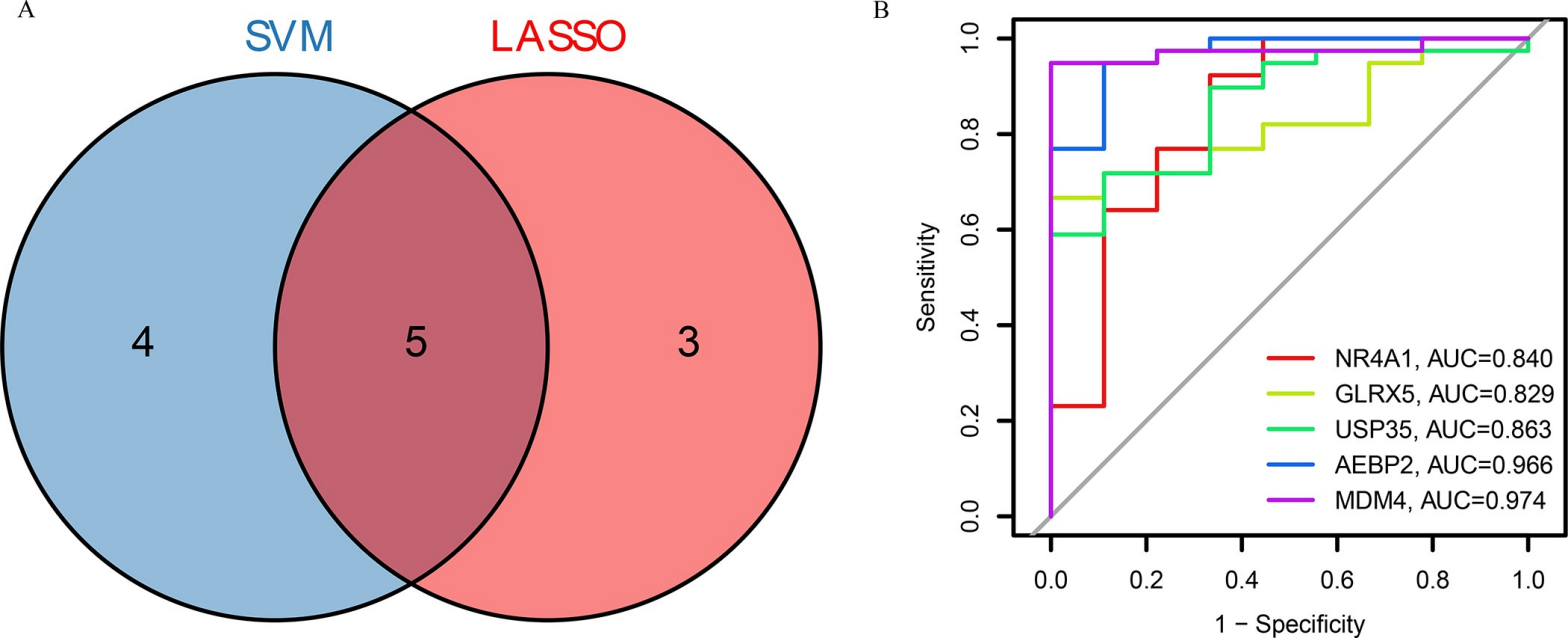
(A) The marker genes obtained from the LASSO and SVM-RFE models. (B) ROC curves for the 5 marker genes.

We aimed to estimate the diagnostic potential of DE-FRGs by comparing diseased patients to healthy individuals. Using four machine learning algorithms (Fig 3A and 3B), we selected LASSO (AUC = 0.951) and SVM (AUC = 0.947) to screen significant DE-FRGs, distinguishing diseased from normal individuals. The LASSO logistic regression algorithm with 10-fold cross-validation identified 8 disease-related features (Fig 3C and 3D), while the SVM-RFE algorithm selected 9 disease-related features. Ultimately, 5 genes (maximal accuracy  = 0.920, Error  = 0.080) were identified as the optimal feature genes (Fig 3E and 3F). *NR4A1*, *GLRX5*, *USP35*, *AEBP2*, and *MDM4* were confirmed as key biomarkers for AKI (Fig 4A). ROC curves for these genes demonstrated AUC values greater than 0.8, indicating high diagnostic accuracy (Fig 4B).

### Immune landscape analysis

**Fig 5:**
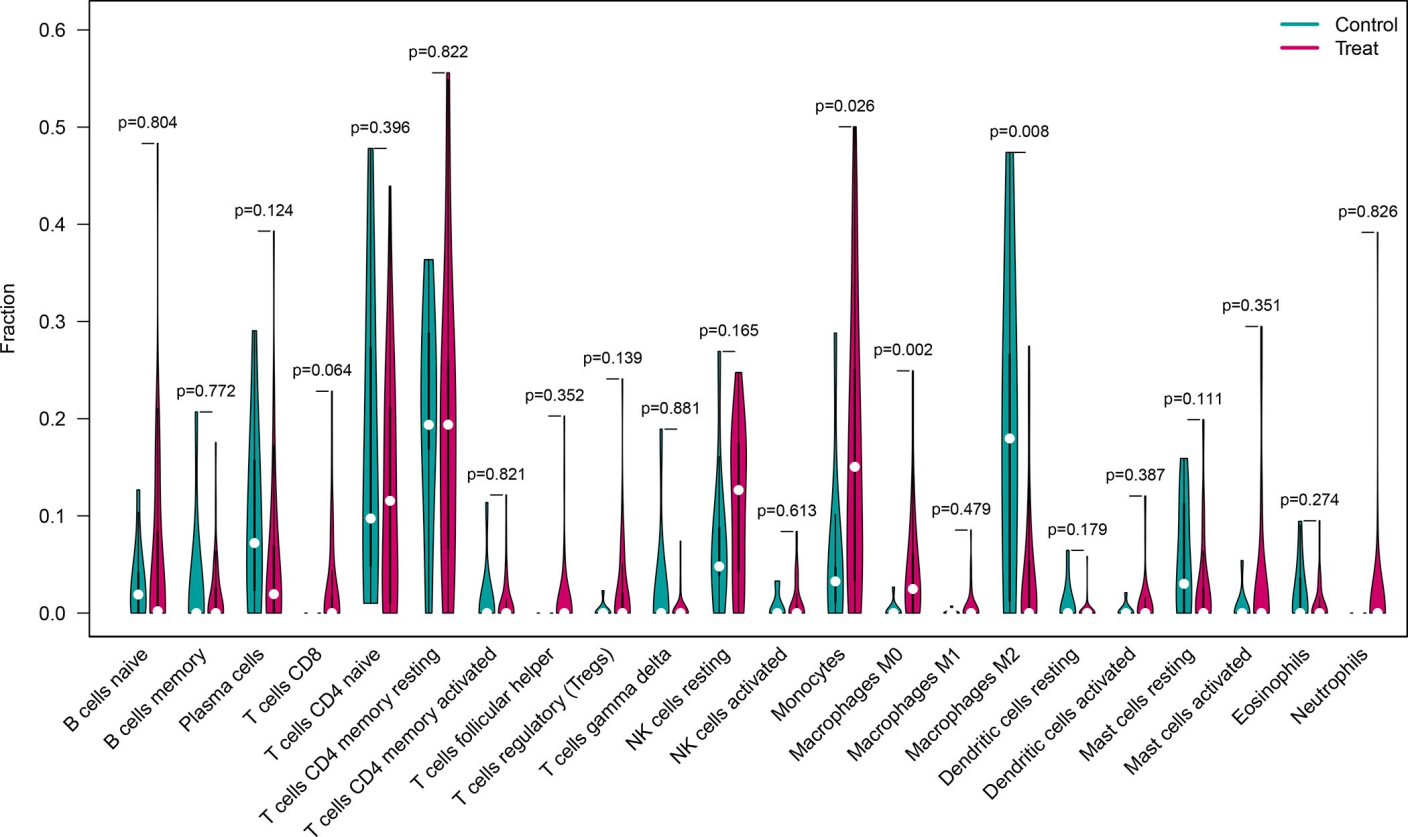
Immune landscape analysis. Key observations include the identification of predominant immune cell types, their relative abundances, and the potential implications for immune response and disease progression.

CIBERSORT analysis revealed differences in the immune microenvironment between diseased and normal samples (Fig 5). M0 macrophages were significantly elevated in AKI samples, while M2 macrophages were higher in normal samples, suggesting a role in the early inflammatory response of AKI.

### qRT−PCR analysis of mRNA levels in tissue samples

**Fig 6:**
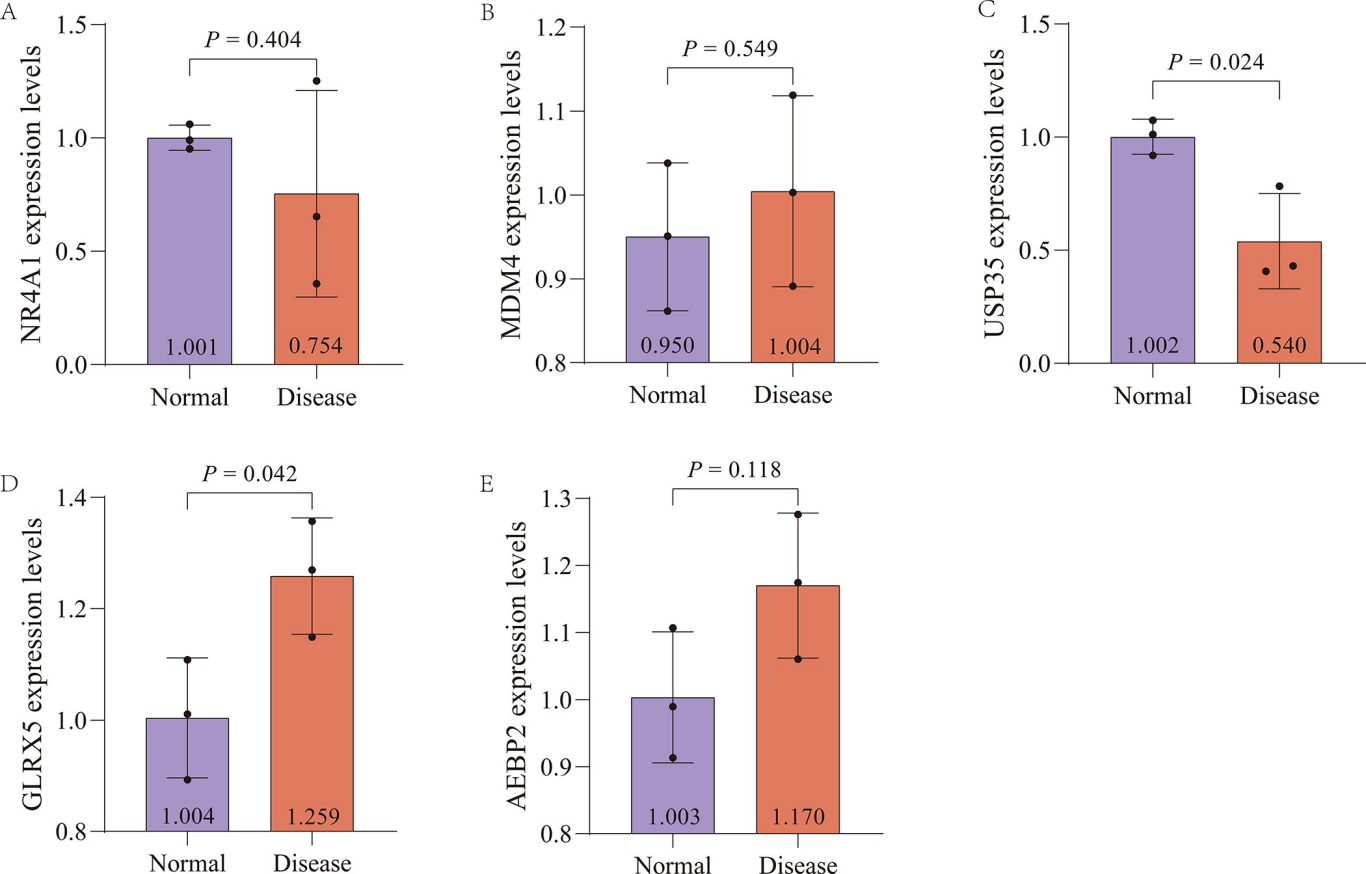
qRT−PCR analysis of mRNA levels in tissue samples. Bar chart showing the relative expression levels of five genes normalized to GAPDH as the reference gene (A: *NR4A1*; B: *MDM4*; C: *USP35*; D: *GLRX5*; E: *AEBP2*). The expression levels were measured using quantitative real-time PCR (qRT-PCR) across different experimental groups. Data are presented as mean ± standard deviation (SD) of three independent experiments.

Real-time qPCR and statistical analysis on ischemia-reperfusion injury mice showed differential expression of marker genes (Fig 6). *NR4A1* expression was lower in disease tissues compared to normal tissues (Fig 6A), while *MDM4* and *AEBP2* expression were higher in disease tissues (Fig 6B–6D). *GLRX5* expression was significantly higher in disease tissues (*P* < 0.05) (Fig 6C), and *USP35* expression was significantly lower in disease tissues (*P* < 0.05) (Fig 6E).

## Discussion

In this study, we investigated the role of FRGs in AKI and identified several key biomarkers with potential diagnostic and therapeutic implications. Our findings revealed that 78 FRGs were differentially expressed between AKI and normal samples, with 27 up-regulated and 51 down-regulated genes. Using lasso and SVM, we identified five critical genes (*NR4A1*, *GLRX5*, *USP35*, *AEBP2*, and *MDM4*) involved in significant signaling pathways such as MAPK, ferroptosis, and p53, which are crucial in cell death and survival mechanisms.

The association between AKI and ferroptosis presents a new perspective in kidney injury research. Recent studies have demonstrated the therapeutic potential of targeting ferroptosis to alleviate kidney injury [19,20]. Among the identified biomarkers, *NR4A1*, *GLRX5*, and *USP35* are categorized as ferroptosis suppressor genes, while *AEBP2* and *MDM4* are considered ferroptosis marker genes. *MDM4*, a cytoplasmic protein, exhibits p53-activating functions in response to DNA damage. Specifically, *MDM4* promotes the phosphorylation of p53 at Ser46, a modification that precedes various p53 activities [21]. Under DNA damage conditions, *MDM4*, predominantly localized in the cytoplasm, can collaborate with p53 to enhance mitochondrial apoptotic activity [22]. GSVA analysis confirmed the activation of the autophagy regulatory pathway in the up-regulated AKI group. Other studies have shown that *MDMX* can immortalize primary mouse embryonic fibroblasts and accelerate the growth of human fibroblasts [23]. The potential of *MDM4* as a therapeutic target for mitigating kidney injury is underscored by its pivotal role in these processes. The high expression of *MDM4* in AKI tissues suggests that targeting this gene could be beneficial for reducing kidney injury and improving patient outcomes.

*AEBP2* is a DNA-binding transcription factor necessary for the modulation of Polycom repressive complex 2 (PRC2) activity, leading to transcriptional repression and gene silencing [24]. *NR4A1*, belonging to the nuclear receptor superfamily, is widely expressed in various cell types and plays a role in regulating inflammatory responses, oxidative stress, and immunity [25,26]. *NR4A1* has been associated with promoting mitochondrial oxidative stress and has been implicated in the development of diabetic nephropathy [27]. Although many of these genes have been extensively studied in cancer biology, their associations with AKI have not been previously reported, indicating the need for further investigations to elucidate their specific roles in AKI pathogenesis.

The MAPK pathway’s role in cell death and survival mechanisms is well-documented. Iron accumulation triggers the activation of the MAPK pathway, leading to neuronal cell death. Suppression of MAPK activation has been shown to reduce cell death, with the pathway inducing both ferroptosis and apoptosis [28]. In AKI, the significant correlation of the MAPK pathway with the identified biomarkers suggests its therapeutic relevance. P53, a well-known tumor suppressor, initiates apoptosis and ferroptosis in response to DNA damage [29]. The inhibitor of the apoptosis-stimulating protein of p53 (iASPP) inhibits p53-induced apoptosis and promotes tumor growth. Overexpression of iASPP induces chemoresistance in human cancer cells [30]. Moreover, iASPP facilitates the accumulation and nuclear translocation of nuclear factor (erythroid-derived 2)-like 2 (Nrf2), which confers cellular protection against oxidative stress-induced by various forms of cell death, including ferroptosis, apoptosis, and autophagy [31,32]. These findings suggest that, along with inducing ferroptosis, the activation of MAPK and p53 signaling pathways may contribute to the generation of reactive oxygen species (ROS), exacerbating cell injury in AKI.

Macrophages are pivotal in the inflammatory response and tissue repair processes [33]. In AKI, macrophages are recruited to the kidney in response to injury signals and can exert both beneficial and detrimental effects [20]. The classification of macrophages into distinct subsets, such as M0, M1 (pro-inflammatory), and M2 (anti-inflammatory and tissue repair), helps in understanding their functional diversity and their contribution to AKI pathophysiology [34]. Analyzing the correlations between immune cells and diagnostic signatures, we observed that M0 macrophages were significantly elevated in AKI samples, while M2 macrophages were significantly higher in normal samples. The increased presence of M0 macrophages in AKI samples indicates their involvement in the early inflammatory response of AKI [35,36]. Understanding the polarization of macrophages into M1 (pro-inflammatory) or M2 (anti-inflammatory and tissue repair) phenotypes can guide the development of targeted interventions. By promoting the M2 phenotype, it may be possible to reduce inflammation and enhance tissue repair in AKI, thus improving patient outcomes. Further research in this area may lead to the identification of novel therapeutic approaches aimed at attenuating inflammation and promoting kidney repair in AKI [37,38].

Based on the obtained results, it is suggested that FRGs hold potential as biomarkers for AKI. The differential expression of these genes provides valuable insights into the underlying mechanisms of AKI and serves as diagnostic markers for the disease. This comprehensive understanding not only seeks to identify better diagnostic markers but also explores promising treatment strategies, paving the way for advancements in AKI management.

Our study, while insightful, has certain limitations that need consideration. The small and uniform sample size from a single dataset may restrict the broader applicability of our findings, requiring larger and more diverse groups for validation. The lack of functional validation experiments means further laboratory and clinical studies are necessary to confirm the roles of identified FRGs in AKI. Additionally, analyzing data from only one-time point may not fully capture the changes in gene expression throughout the progression of AKI, indicating a need for longitudinal studies. Despite these limitations, our study offers significant strengths. We identified novel biomarkers (*NR4A1*, *GLRX5*, *USP35*, *AEBP2*, and *MDM4*) with high diagnostic potential and provided comprehensive insights into the mechanisms and immune responses in AKI. Using multiple analytical methods strengthens our findings, and the proposed strategies targeting ferroptosis and iron regulation present innovative pathways for future research and treatment development in AKI.

## Conclusions

In conclusion, our study highlights the critical role of FRGs in AKI. We identified 78 differentially expressed FRGs, with *NR4A1*, *GLRX5*, *USP35*, *AEBP2*, and *MDM4* emerging as key biomarkers. These genes are involved in essential pathways such as MAPK, ferroptosis, and p53, highlighting their potential in cell survival and death mechanisms. Targeting ferroptosis and regulating iron homeostasis, combined with modulating macrophage polarization, offers innovative strategies for AKI treatment. Our findings not only enhance early diagnosis but also pave the way for new therapeutic interventions, promising significant advancements in AKI management. Future research should validate these biomarkers in clinical settings and explore their therapeutic potential further.

## Supporting information

S1 Fig Single-gene GSEA-KEGG pathway analysis (A: *AEBP2*; B: *MDM4*; C: *USP35*; D: *GLRX5*; E: *NR4A1*). (TIF)

S1 Table The gene list of 254 ferroptosis-related genes. This table provides a comprehensive list of genes associated with ferroptosis, which were used in the analysis.
(DOCX)

S2 Table The table of 78 differentially expressed genes (DEGs). This table includes the genes identified as differentially expressed in acute kidney injury (AKI) samples compared to normal samples, along with their fold changes and statistical significance.
(DOCX)

## Abbreviations

AKI: Acute kidney injury
FRGs: ferroptosis-related genes
DE-FRGs: Differentially expressed ferroptosis-related genes
GEO: Gene Expression Omnibus
DEGs: Differentially expressed genes
sCr: serum creatinine concentration
FER-1: Ferrostatin-1
logFC: log Fold Change
LASSO: least absolute shrinkage and selection operator
TCA: tricarboxylic acid
ROS: reactive oxygen species
FDR: false discovery rate
GO: Gene ontology
BP: biological processes
MF: molecular function
CC: cellular components
KEGG: Kyoto Encyclopedia of Genes and Genomes
PPI: protein-protein interaction
AUC: Area under the curve
ROC: Receiver operating characteristic
C-index: concordance index
TIMER: tumor immune estimation resource
iASPP: inhibitor of apoptosis-stimulating protein of p53
EMLA: Eutectic Mixture of Local Anesthetics
IR: ischemia-reperfusion injury
