# Supplementary figures and images for: Exploring the role of ferroptosis-related genes as biomarkers in acute kidney injury
Source: PLoS One. 2024 Jul 23;19(7):e0307472. doi: 10.1371/journal.pone.0307472 (PMC11265698; doi:10.1371/journal.pone.0307472)

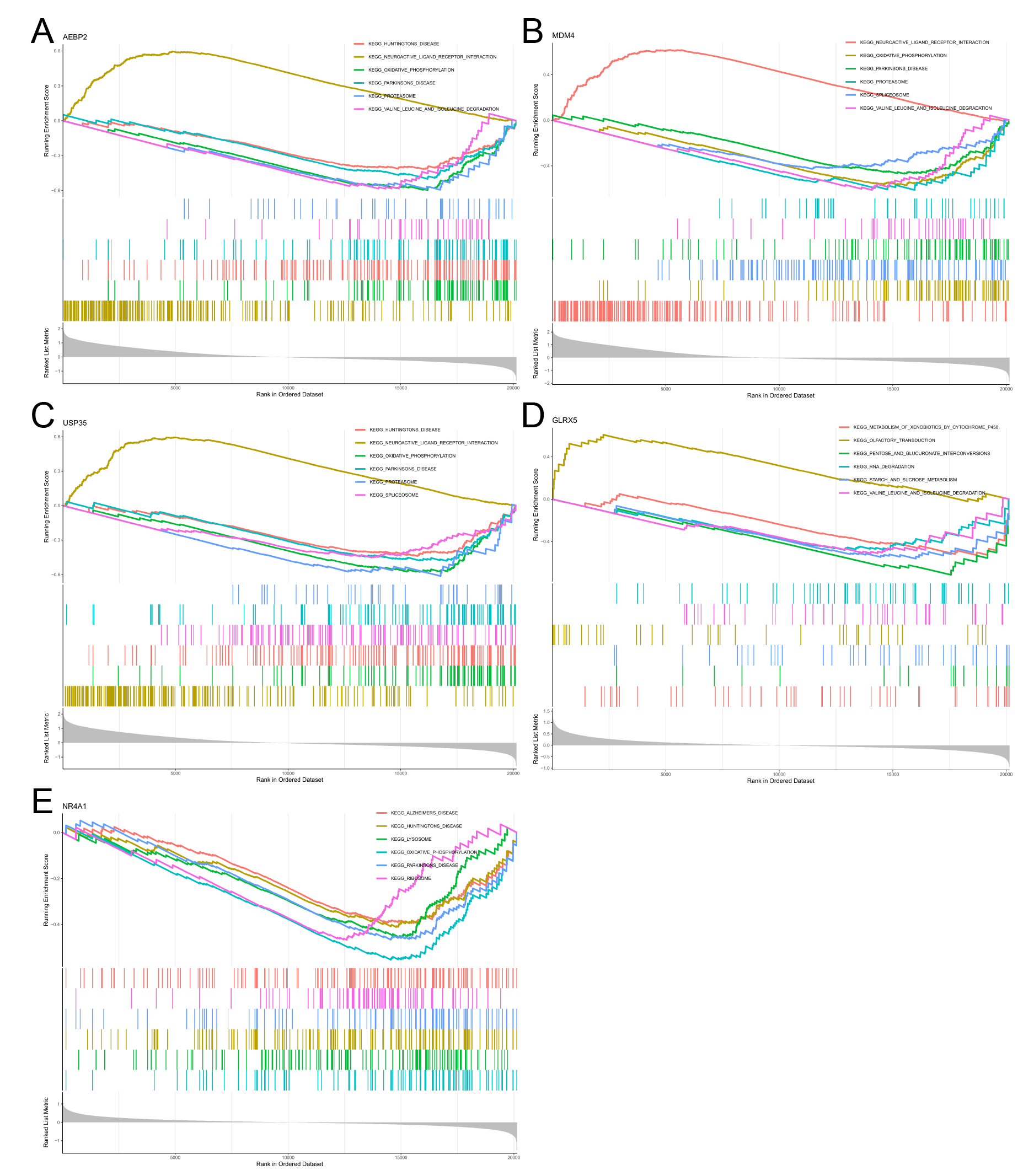

Supplement: S1 Fig — (TIF) [file pone.0307472.s001.tif]
