# Supplementary material for: Exploring the role of ferroptosis-related genes as biomarkers in acute kidney injury
Source: PLoS One. 2024 Jul 23;19(7):e0307472. doi: 10.1371/journal.pone.0307472 (PMC11265698; doi:10.1371/journal.pone.0307472)
Supplement: S2 Table — This table includes the genes identified as differentially expressed in acute kidney injury (AKI) samples compared to normal samples, along with their fold changes and statistical significance. (DOCX) [file pone.0307472.s003.docx]

Supplementary Table 2| The table of 78 DEGs.

| id | conMean | treatMean | log2FoldChange | pValue | direction |
| --- | --- | --- | --- | --- | --- |
| AKR1C1 | 6.66 | 4.52 | -2.14 | 0.01 | Down |
| NQO1 | 7.79 | 6.16 | -1.62 | 0.02 | Down |
| STAT3 | 10.80 | 10.23 | -0.58 | 0.05 | Down |
| NFS1 | 6.78 | 5.23 | -1.55 | 0.04 | Down |
| ENPP2 | 8.69 | 7.24 | -1.44 | 0.00 | Down |
| FH | 7.69 | 5.59 | -2.10 | 0.02 | Down |
| JUN | 10.09 | 9.10 | -0.99 | 0.02 | Down |
| ZFP36 | 8.44 | 7.04 | -1.40 | 0.03 | Down |
| DAZAP1 | 8.27 | 8.93 | 0.66 | 0.03 | Up |
| SLC16A1 | 7.36 | 5.02 | -2.33 | 0.01 | Down |
| NR4A1 | 9.35 | 6.86 | -2.49 | 0.00 | Down |
| ATF2 | 7.76 | 6.72 | -1.04 | 0.05 | Down |
| STK11 | 8.07 | 9.36 | 1.29 | 0.00 | Up |
| CDH1 | 9.51 | 7.56 | -1.95 | 0.01 | Down |
| NEDD4L | 10.17 | 9.54 | -0.64 | 0.03 | Down |
| GLRX5 | 5.49 | 7.98 | 2.49 | 0.00 | Up |
| NCOA3 | 9.51 | 8.80 | -0.71 | 0.05 | Down |
| ARF6 | 8.23 | 9.70 | 1.47 | 0.03 | Up |
| AHCY | 8.16 | 7.27 | -0.89 | 0.04 | Down |
| PPARA | 9.59 | 8.30 | -1.29 | 0.01 | Down |
| NEDD4 | 8.37 | 7.34 | -1.04 | 0.02 | Down |
| USP35 | 7.09 | 9.54 | 2.46 | 0.00 | Up |
| PDSS2 | 6.94 | 5.14 | -1.80 | 0.01 | Down |
| GOT1 | 8.66 | 6.20 | -2.46 | 0.00 | Down |
| SIRT1 | 8.16 | 8.67 | 0.52 | 0.04 | Up |
| TYRO3 | 7.68 | 6.35 | -1.33 | 0.05 | Down |
| VCP | 10.38 | 9.74 | -0.64 | 0.05 | Down |
| RBMS1 | 8.98 | 9.91 | 0.93 | 0.03 | Up |
| MPC1 | 7.73 | 6.50 | -1.23 | 0.04 | Down |
| SRSF9 | 7.51 | 8.91 | 1.41 | 0.04 | Up |
| MIR27A | 0.54 | 2.04 | 1.49 | 0.01 | Up |
| PDK4 | 9.71 | 7.69 | -2.02 | 0.03 | Down |
| PTPN18 | 8.24 | 8.83 | 0.58 | 0.05 | Up |
| GALNT14 | 9.36 | 7.75 | -1.62 | 0.04 | Down |
| TERT | 1.80 | 3.72 | 1.91 | 0.02 | Up |
| ACSF2 | 9.81 | 8.24 | -1.56 | 0.04 | Down |
| NOX4 | 8.35 | 6.34 | -2.00 | 0.00 | Down |
| HRAS | 3.65 | 5.64 | 1.99 | 0.04 | Up |
| TFR2 | 2.72 | 4.59 | 1.88 | 0.03 | Up |
| ALOX15 | 1.68 | 3.03 | 1.36 | 0.05 | Up |
| ACO1 | 9.31 | 7.77 | -1.55 | 0.00 | Down |
| EGFR | 10.08 | 9.36 | -0.73 | 0.00 | Down |
| DPP4 | 9.25 | 6.99 | -2.26 | 0.00 | Down |
| PEBP1 | 8.81 | 7.23 | -1.59 | 0.04 | Down |
| ELAVL1 | 8.27 | 7.16 | -1.11 | 0.00 | Down |
| BAP1 | 8.99 | 9.25 | 0.26 | 0.03 | Up |
| EPAS1 | 11.16 | 10.29 | -0.88 | 0.03 | Down |
| HILPDA | 3.55 | 5.08 | 1.53 | 0.04 | Up |
| ATF3 | 7.95 | 6.13 | -1.82 | 0.01 | Down |
| YY1AP1 | 9.25 | 10.14 | 0.89 | 0.00 | Up |
| CD82 | 5.73 | 7.74 | 2.00 | 0.00 | Up |
| AQP3 | 8.01 | 5.73 | -2.28 | 0.03 | Down |
| AQP8 | 0.67 | 2.55 | 1.88 | 0.00 | Up |
| OSBPL9 | 9.49 | 8.29 | -1.20 | 0.04 | Down |
| EPT1 | 7.87 | 6.81 | -1.07 | 0.05 | Down |
| AEBP2 | 8.66 | 11.85 | 3.19 | 0.00 | Up |
| FAR1 | 9.09 | 7.99 | -1.10 | 0.02 | Down |
| SMAD7 | 7.87 | 8.67 | 0.80 | 0.01 | Up |
| MTCH1 | 9.09 | 8.21 | -0.89 | 0.01 | Down |
| ACADSB | 8.17 | 6.50 | -1.67 | 0.04 | Down |
| SLC39A14 | 8.65 | 6.96 | -1.69 | 0.01 | Down |
| SLC11A2 | 8.71 | 7.60 | -1.11 | 0.04 | Down |
| TGFB1 | 8.23 | 9.01 | 0.78 | 0.01 | Up |
| SNCA | 6.08 | 4.01 | -2.07 | 0.02 | Down |
| MDM2 | 8.98 | 9.93 | 0.95 | 0.01 | Up |
| MDM4 | 9.58 | 11.39 | 1.82 | 0.00 | Up |
| DLD | 8.84 | 7.25 | -1.59 | 0.03 | Down |
| LGMN | 8.88 | 7.43 | -1.46 | 0.02 | Down |
| YTHDC2 | 8.97 | 7.83 | -1.14 | 0.03 | Down |
| TRIM46 | 2.31 | 4.91 | 2.59 | 0.00 | Up |
| ACSL1 | 10.36 | 8.94 | -1.42 | 0.01 | Down |
| DPEP1 | 8.89 | 5.76 | -3.13 | 0.00 | Down |
| GJA1 | 6.78 | 5.01 | -1.77 | 0.04 | Down |
| CIRBP | 10.12 | 10.62 | 0.49 | 0.04 | Up |
| TRIM26 | 5.59 | 7.56 | 1.97 | 0.03 | Up |
| NDRG1 | 10.79 | 9.52 | -1.27 | 0.01 | Down |
| LIFR | 10.11 | 8.75 | -1.36 | 0.00 | Down |
| MIB1 | 9.54 | 8.72 | -0.82 | 0.01 | Down |
